# Supplementary figures and images for: Vaccination of yearling horses against poly-N-acetyl glucosamine fails to protect against infection with Streptococcus equi subspecies equi
Source: PLoS One. 2020 Oct 15;15(10):e0240479. doi: 10.1371/journal.pone.0240479 (PMC7561144; doi:10.1371/journal.pone.0240479)

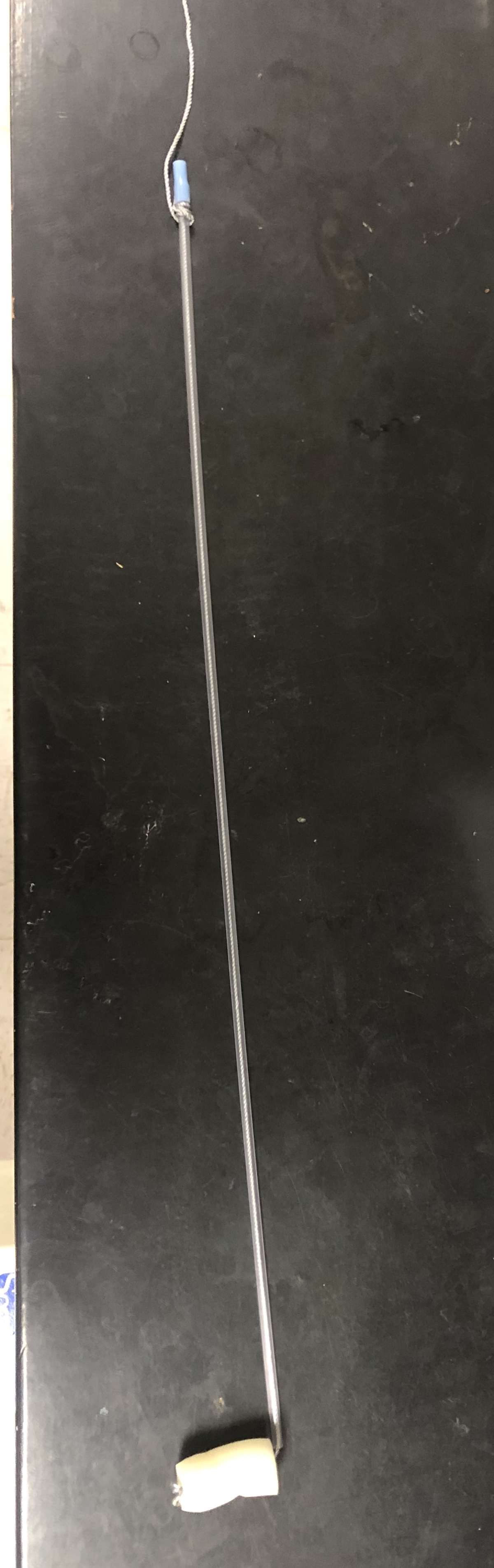

Supplement: S1 Fig — Sterile absorbent foam plugs (Identi-Plugs Size B2, Jaece Industries, North Tonawanda, NY, USA) were tethered to a string and were inserted into the nasal passages of each horse to a depth of approximately 5 to 8 cm using a plastic equine insemination catheter as an introducer. (JPG) [file pone.0240479.s001.jpg]

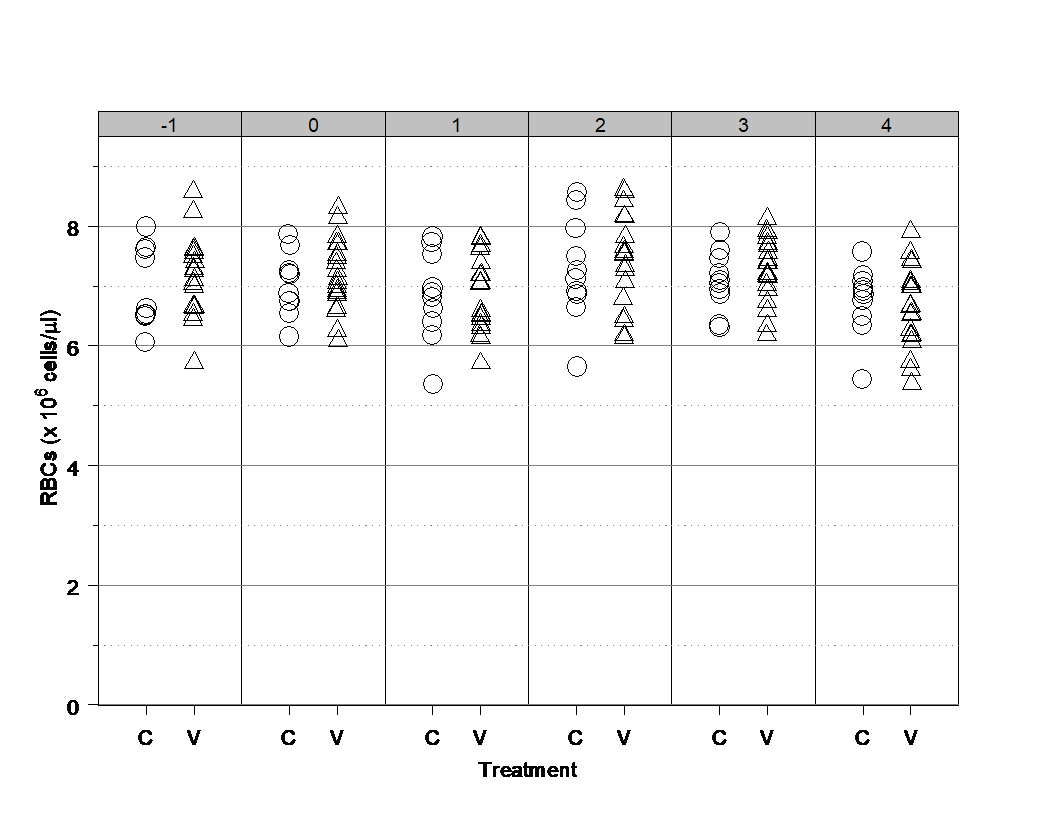

Supplement: S2 Fig — The 6 vertical panels represent weeks, with week -1 representing sampling 1 week prior to vaccination, and week 0 representing the day of vaccination, followed by weeks 1, 2, 3, and 4 after vaccination. There were no significant differences among groups within times, or within group over time. (TIF) [file pone.0240479.s002.tif]

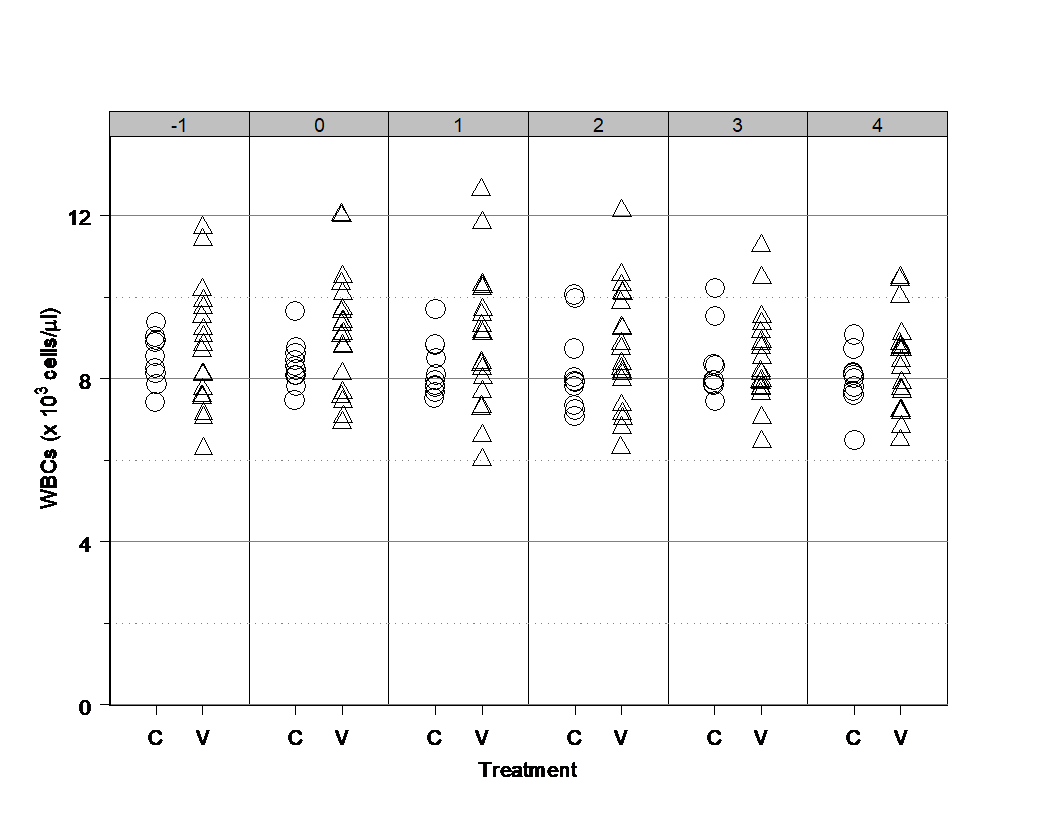

Supplement: S3 Fig — The 6 vertical panels represent weeks, with week -1 representing sampling 1 week prior to vaccination, and week 0 representing the day of vaccination, followed by weeks 1, 2, 3, and 4 after vaccination. There were no significant differences among groups within times, or within group over time. (TIF) [file pone.0240479.s003.tif]

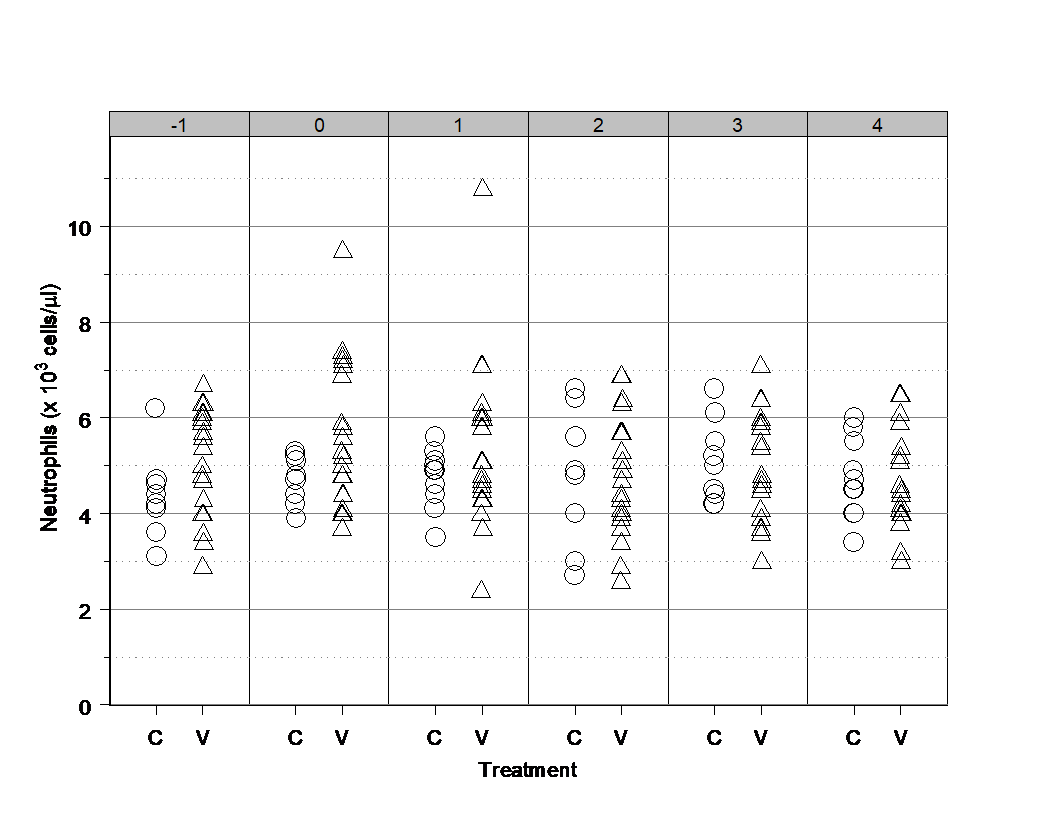

Supplement: S4 Fig — The 6 vertical panels represent weeks, with week -1 representing sampling 1 week prior to vaccination, and week 0 representing the day of vaccination, followed by weeks 1, 2, 3, and 4 after vaccination. There were no significant differences among groups within times, or within group over time. (TIF) [file pone.0240479.s004.tif]

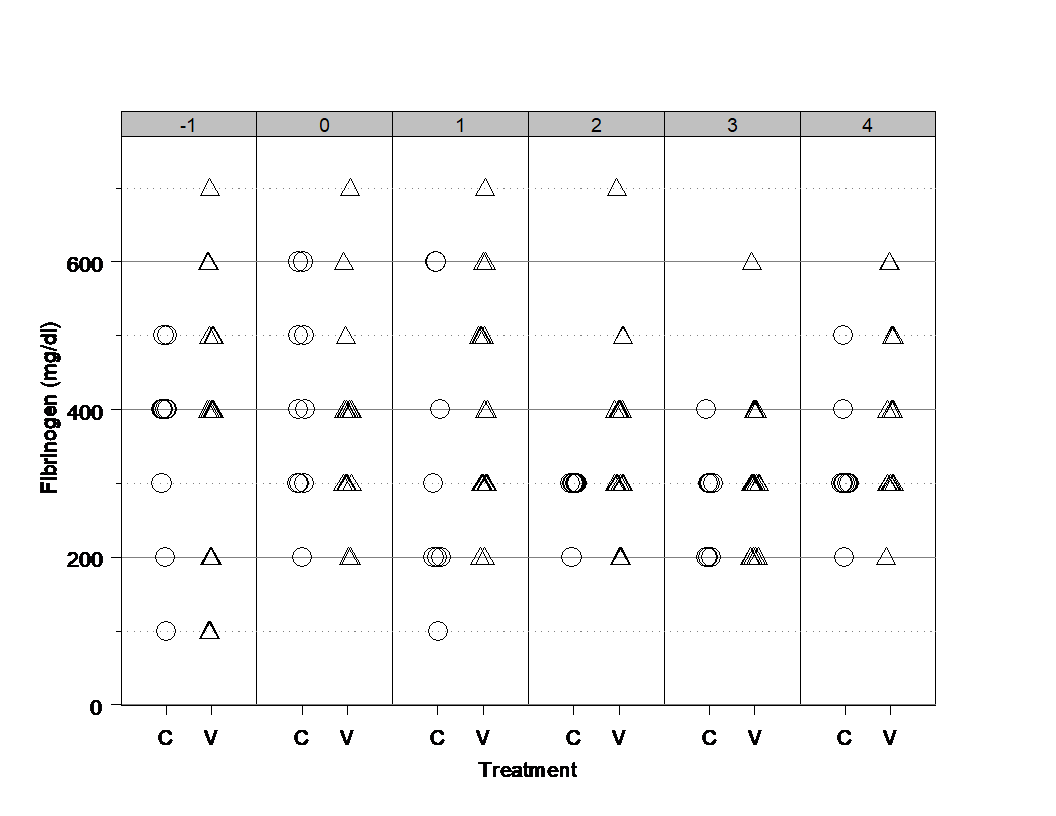

Supplement: S5 Fig — The 6 vertical panels represent weeks, with week -1 representing sampling 1 week prior to vaccination, and week 0 representing the day of vaccination, followed by weeks 1, 2, 3, and 4 after vaccination. There were no significant differences among groups within times, or within group over time. (TIF) [file pone.0240479.s005.tif]

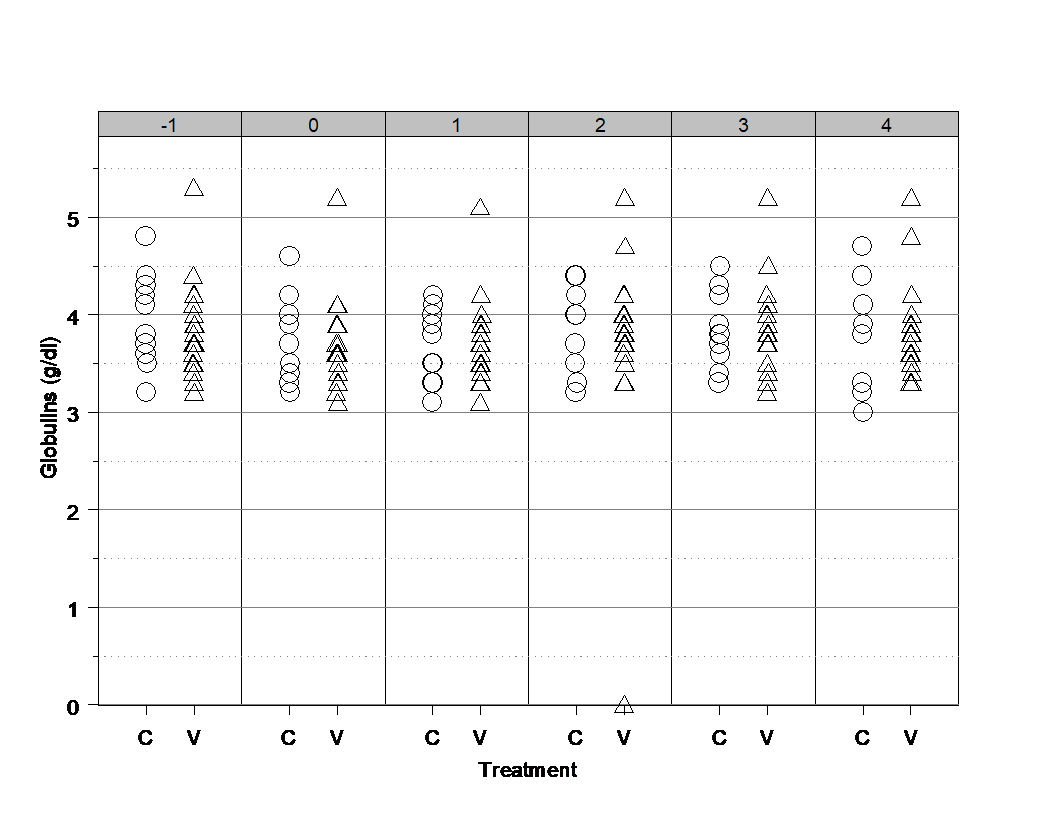

Supplement: S6 Fig — The 6 vertical panels represent weeks, with week -1 representing sampling 1 week prior to vaccination, and week 0 representing the day of vaccination, followed by weeks 1, 2, 3, and 4 after vaccination. There were no significant differences among groups within times, or within group over time. (TIF) [file pone.0240479.s006.tif]

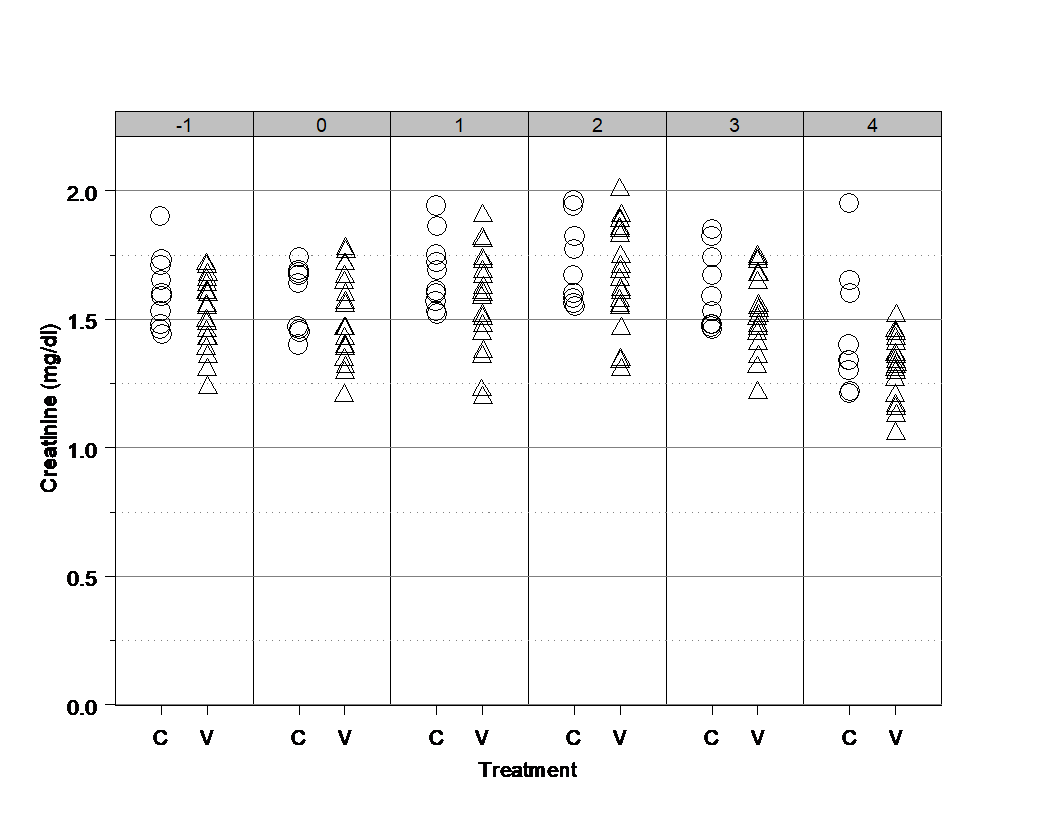

Supplement: S7 Fig — The 6 vertical panels represent weeks, with week -1 representing sampling 1 week prior to vaccination, and week 0 representing the day of vaccination, followed by weeks 1, 2, 3, and 4 after vaccination. There were no significant differences among groups within times, or within group over time. (TIF) [file pone.0240479.s007.tif]

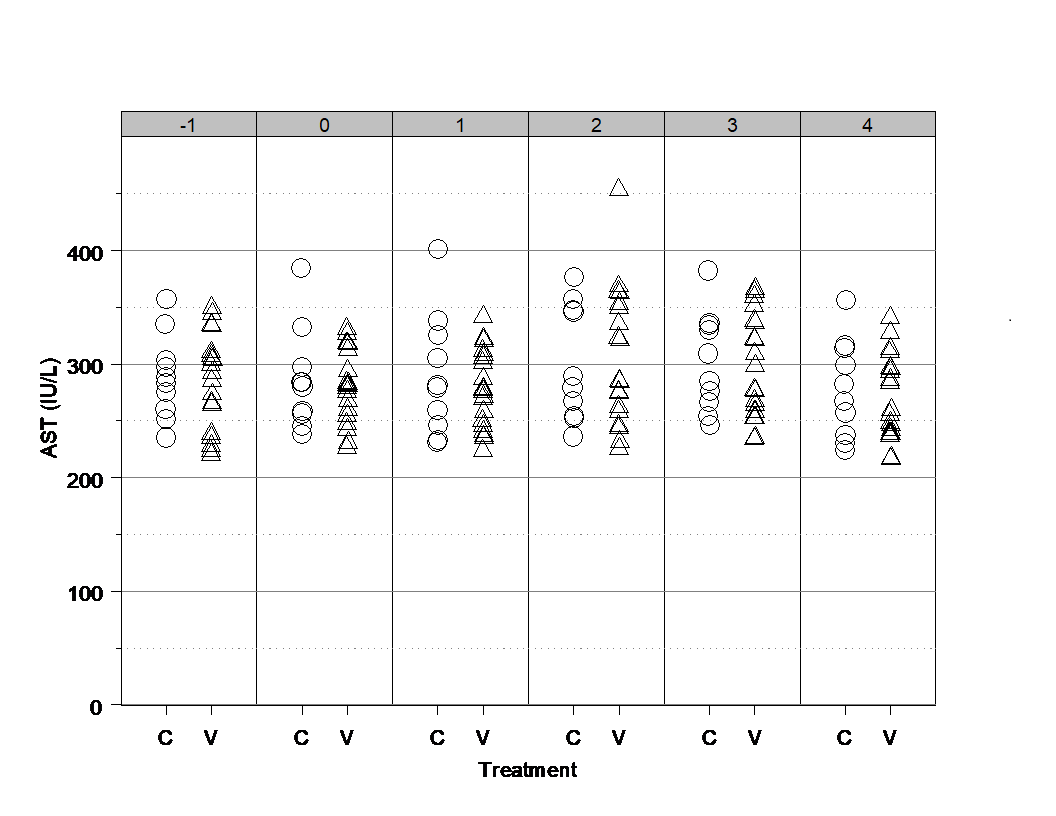

Supplement: S8 Fig — The 6 vertical panels represent weeks, with week -1 representing sampling 1 week prior to vaccination, and week 0 representing the day of vaccination, followed by weeks 1, 2, 3, and 4 after vaccination. There were no significant differences among groups within times, or within group over time. (TIF) [file pone.0240479.s008.tif]
